# Ethnic variations in sexual partnerships and mixing, and their association with STI diagnosis: findings from a cross-sectional biobehavioural survey of attendees of sexual health clinics across England

**DOI:** 10.1136/sextrans-2018-053739

**Published:** 2019-08-17

**Authors:** Catherine RH Aicken, Sonali Wayal, Paula Blomquist, Stella Fabiane, Makeda Gerressu, Gwenda Hughes, Catherine H Mercer

**Affiliations:** 1 Institute for Global Health, University College London, London, UK; 2 School of Health Sciences, University of Brighton, Brighton, UK; 3 Centre for Infectious Disease Surveillance and Control, Public Health England, London, UK; 4 MRC Clinical Trials Unit, University College London, London, UK

**Keywords:** ethnicity, sexual behaviour, epidemiology (general), infectious diseases

## Abstract

**Objectives:**

Ethnic differences in partnership types and sexual mixing patterns may contribute to elevated STI diagnosis rates among England’s Black Caribbean (BC) population. We examined the differences between BC and White British/Irish (WBI) sexual health clinic (SHC) attendees’ reported partnerships and sexual mixing, and whether these differences could explain ethnic inequalities in STI, focusing on attendees reporting only opposite-sex partners (past year).

**Methods:**

We surveyed attendees at 16 SHCs across England (May to September 2016), and linked their survey responses to routinely collected data on diagnoses of bacterial STI or trichomoniasis ±6 weeks of clinic attendance (‘acute STI’). Behaviourally-heterosexual BC and WBI attendees (n=1790) reported details about their ≤3 most recent opposite-sex partners (past 3 months, n=2503). We compared BC and WBI attendees’ reported partnerships and mixing, in gender-stratified analyses, and used multivariable logistic regression to examine whether they independently explained differences in acute STI.

**Results:**

We observed differences by ethnic group. BC women’s partnerships were more likely than WBI women’s partnerships to involve age-mixing (≥5 years age difference; 31.6% vs 25.5% partnerships, p=0.013); BC men’s partnerships were more often ‘uncommitted regular’ (35.4% vs 20.7%) and less often casual (38.5% vs 53.1%) than WBI men’s partnerships (p<0.001). Acute STI was higher among BC women than WBI women (OR: 2.29, 95% CI 1.24 to 4.21), with no difference among men. This difference was unaffected by partnerships and mixing: BC women compared with WBI women adjusted OR: 2.31 (95% CI 1.30 to 4.09) after adjusting for age and partner numbers; 2.15 (95% CI 1.07 to 4.31) after additionally adjusting for age-mixing, ethnic-mixing and recent partnership type(s).

**Conclusion:**

We found that differences in sexual partnerships and mixing do not appear to explain elevated risk of acute STI diagnosis among behaviourally-heterosexual BC women SHC attendees, but this may reflect the measures used. Better characterisation of ‘high transmission networks’ is needed, to improve our understanding of influences beyond the individual level, as part of endeavours to reduce population-level STI transmission.

## Introduction

Ethnic inequalities in STI have been repeatedly observed in Britain^1–3^ (and elsewhere^4 5^) but their causes remain unclear. In the general population, and among sexual health clinic (SHC) attendees, STI diagnoses are more common among people of Black ethnicities,^1 6–8^ particularly bacterial STI^9^ and trichomoniasis^10^ diagnosis rates among Black Caribbean (BC) people,^3^ for example, 378 gonorrhoea and 224 trichomoniasis diagnoses per 100 000,^11^ vs 67 and 8 respectively among people of White ethnicity, in England.^12^ 1.1% of England and Wales’ population self-defines as BC (n=594 825).^13^

Understanding the factors which drive ethnic inequalities in STIs is essential to develop appropriate interventions. Proposed ‘explanations’ include behavioural differences between ethnic groups, in partner numbers, concurrency^14^ and contexts of condom use.^15^ Ethnic differences in STI diagnoses remain, but are attenuated, after accounting for individual characteristics and behaviours such as age, deprivation^3 9^ and partner numbers.^3^ Characteristics of individuals’ *partner(ship)s* may also influence the likelihood of STI, and therefore inequalities in STI across the population. National probability survey data show that people’s partner(ship) type(s) influence their likelihood of reporting recent STI diagnoses, independent of age and partner numbers.^16^ Heterosexual partnerships, and particularly steady and cohabiting partnerships, tend to be assortative, that is, people tend to share characteristics with their partners,^17 18^ while *dis*assortative sexual mixing is more common among casual partnerships.^18^ Age-disassortative heterosexual partnerships, particularly where women are younger, are less likely to involve condom use,^18–20^ and are associated with reporting recent STI diagnoses, among women.^18^ Mathematical modelling studies demonstrate that sexual mixing patterns can contribute to establishing and perpetuating differences in STI incidence between population groups, thus hindering or facilitating transmission through the population as a whole.^21 22^ Theoretically, assortative ethnic-mixing within a high STI prevalence group would tend to increase STI prevalence within this group; conversely disassortative partnerships may ‘bridge’ lower and higher prevalence populations. We therefore considered that sexual ‘mixing’ (by age and ethnic group), and partnership type, may help explain the inequitable distribution of STI.

Our study complements findings from probability surveys of Britain’s general population, among which partner change rates are relatively low,^23^ and BC men (but not women) report slightly larger partner numbers than their White British counterparts (after accounting for age distribution differences).^3^ By focusing on SHC attendees, we sample people at elevated STI risk, who are under-represented in probability samples of the general population,^24^ contribute disproportionately to STI transmission, are more likely to experience STI diagnosis, and report higher partner numbers and concurrency than non-attendees.^25 26^ Furthermore, their engagement with services makes them potential candidates for intervention.

This study aims to describe sexual partnership type(s), age-mixing and ethnic-mixing among behaviourally-heterosexual SHC attendees, focusing on those of BC ethnicity, and then examines whether any differences explain BC men’s and women’s greater risk of bacterial STIs and trichomoniasis, relative to people of White British/Irish (WBI) ethnicity (the UK’s ethnic majority). Additionally, we make comparisons by gender within these ethnic groups, reflecting well-established gender differences in reported sexual behaviour^23^ and the ‘sexual scripts’ shaping these behaviours.^27^

## Methods

We developed a web-based patient survey (online supplementary appendix survey), as part of a Bio-Behavioural Enhanced Surveillance Tool (BBEST).^28^ Between May and September 2016, the survey was offered to people (of all ethnicities) attending 16 SHCs across England which were purposively selected (based on GUMCAD, England’s STI surveillance programme, 2014) for their high proportions of BC attendees (7%–32% of clinic attendances). Eligible people were aged ≥15 years, and reported having had sex in the previous 12 months. Participants completed the survey in clinic or elsewhere on their own devices. We linked participants’ data, with consent, to an extract of their routinely collected clinical data (prepared for GUMCAD). Of 3986 survey completers, 91% consented to linkage (3611); of these, linkage was achieved for 91% (3284).

10.1136/sextrans-2018-053739.supp3 Supplementary data

In the current study, we restricted our sample to participants identifying as male or female, and reporting only opposite-sex partners within the past 12 months (hereon, ‘behaviourally-heterosexual’). We focused on SHC attendees self-identifying as BC, and WBI attendees (the ethnic majority) as a comparator (total: 1790). To maximise statistical power, we used this full sample where possible (tables 1–3), using linked data only for subanalyses where STI diagnosis was an outcome (table 4). (Findings for other ethnicities are found in online supplementary tables 1–4.)

10.1136/sextrans-2018-053739.supp1 Supplementary data

### Participant-level data

The survey included questions on participants’ sociodemographics and recent sexual behaviour, including numbers and genders of partners, concurrency (overlapping sexual partnerships) and participants’ current partnership(s) status (casual partner(s) only, committed partner(s) only, casual *and* committed, or none). Bacterial STI(s) and/or trichomoniasis diagnoses from 6 weeks before to 6 weeks after clinic attendance (hereon, ‘acute STI’) were obtained from clinical data.

### Partnership-level data

The survey asked about attendees’ (up to) three most recent partner(s), within the 3 months before their SHC attendance. Details included each partner’s: age, ethnic group, how they met, how long ago first and most recent sex occurred, and *at most recent sex with the partner*: condom use, whether the participant expected to have sex with the partner again (a proxy for ongoing/ended partnerships) and partnership type. We created three categories from partnership type response options (online supplementary web-appendix survey): ‘committed’, ‘uncommitted regular’ and ‘casual’. We defined ‘age-mixing’ as sex between people with ≥5 years’ age difference,^29^ and ‘ethnic-mixing’ as sex between people of different ethnic groups.

In our study’s sample, 94.6% of women and 86.6% of men reported ≤3 sexual partners within the past 3 months, meaning that these *partnership*-level data are complete (in theory) for the large majority of participants. This corresponds to an estimated 79.5% of female participants’ *partners* and 64.0% of male participants’ *partners*, as data were not collected on fourth and higher order partners.

### Analyses

We used Stata V.14 (StataCorp), and accounted for clustering of participants by clinic. We did not additionally account for clustering of partnership-level data by participant, as it is generally preferable to account only for the highest level of clustering.^30^

#### Univariate analyses of participant/partnership-level data

We used χ^2^ tests to compare BC and WBI attendees in univariate gender-stratified analyses, and to compare men and women in univariate analyses stratified by ethnic group. We then created a *partnership*-level data set, using details participants provided about their recent partner(ship)s. We used χ^2^ tests to compare partnerships reported by BC and WBI attendees in analyses stratified by participants’ gender, and to compare men’s and women’s partnerships in analyses stratified by participants’ ethnic group.

#### Multivariable analysis

In the participant-level data set, we used multivariable logistic regression models to examine whether accounting for ethnic and gender differences in sexual mixing and partnership type explained ethnic variations in STI diagnoses. First, we adjusted for participant’s age and partner numbers (past 3 months), as potential confounders.^25^ Then, we adjusted additionally for partnership and mixing using binary variables derived from partnership-level data (on the most recent ≤3 partners, within the past 3 months): any committed partners, any uncommitted regular partners, any casual partners, any age-mixing and any ethnic-mixing.

## Results

### Variations in sociodemographic characteristics and sexual behaviours

Men of BC and WBI ethnicities had a median age of 27 years (table 1). BC men attendees were less likely than WBI men attendees to be educated beyond General Certificate of Secondary Education (GCSE) equivalent, or to be employed. We observed ethnic differences in men’s current partnership(s) status, for example, 7.4% of BC men reported both committed and casual partnerships, while 2.6% of WBI men reported this. Smaller proportions of BC men than WBI men reported: only committed partnership(s) (33.5% and 38.6%, respectively) and no current partnerships (17.6% and 20.1%, respectively). Despite reporting similar numbers of partners within the previous year, BC men were more likely than WBI men to report higher partner numbers within the previous 3 months. Among attendees reporting more than one partner (past year), BC men were more likely than WBI men to report concurrency (57.2% and 50.6%, respectively).

**Table 1:** Variations in the prevalence of reported number and types of sexual partners, by ethnic group and gender

	Men		P for ethnic difference among men	Women		P for ethnic difference among women	P for gender differences	
	Black Caribbean	White British/Irish		Black Caribbean	White British/Irish		Among BC attendees	Among WBI attendees
Denominator (participants)*	182	426		390	792			
**Sociodemographics**								
Age (median, IQR)	27 (22–33)	27 (24–31)		26 (22–31)	25 (21–30)			
15–19	11.0%	4.9%	0.098	12.8%	10.1%	0.327	0.318	0.009
20–24	25.8%	23.9%		32.8%	36.9%			
25–34	42.3%	54.2%		37.4%	40.5%			
35–44	11.0%	12.0%		10.3%	8.3%			
45+	9.9%	4.9%		6.7%	4.2%			
Education above GCSEs, or equivalent†	65.9%	82.2%	0.010	78.8%	84.8%	0.015	0.001	0.081
In employment	71.7%	84.0%	0.004	71.1%	71.3%	0.946	0.850	0.001
**Current partnership(s) status**			0.044			0.167	<0.001	0.009
Committed sexual partnership(s) only	33.5%	38.6%		52.3%	50.1%			
Casual sexual partnership(s) only	41.5%	38.6%		31.1%	28.9%			
Both committed and casual sexual partnerships	7.4%	2.6%		1.8%	1.8%			
No current sexual partnership	17.6%	20.1%		14.8%	19.2%			
**Sexual partners, past year**								
Partners, n			0.160			0.002	<0.001	<0.001
1	16.1%	25.4%		51.0%	42.3%			
2	23.0%	15.9%		24.2%	19.3%			
3–4	23.6%	26.1%		17.6%	23.0%			
5–9	24.7%	21.2%		6.1%	12.6%			
10+	12.6%	11.5%		1.1%	2.8%			
New partners, n			0.053			<0.001	<0.001	<0.001
0	8.9%	3.2%		14.6%	4.8%			
1	34.9%	36.7%		64.9%	53.7%			
2+	56.2%	60.1%		20.5%	41.5%			
Overlapping (concurrent) partnerships,‡ *among those reporting two or more partners in the past year*			0.023			0.176	<0.001	<0.001
*Denominator (participants)§*	*145*	*310*		*192*	*453*			
No	33.1%	43.5%		55.2%	58.7%			
Yes	57.2%	50.6%		41.7%	39.7%			
Don't remember	9.7%	5.8%		3.1%	1.5%			
**Sexual partners, past 3 months, n**			0.029			0.039	<0.001	<0.001
0	7.3%	8.8%		11.3%	6.9%			
1	32.6%	43.4%		66.5%	63.6%			
2	28.1%	23.6%		15.0%	17.8%			
3	15.2%	12.9%		4.0%	5.5%			
4+	16.9%	11.2%		3.2%	6.3%			

*Denominators: participants (SHC attendees) identifying as male, and who reported only female partners in the past year, and participants identifying as female who reported only male partners in the past year. For categorical outcome variables, Pearson χ^2^ tests were used to calculate p values.

†GCSE: General Certificate of Secondary Education, exams typically taken by age 16 in England.

‡From a direct question about overlapping partnerships.

§Among participants reporting two or more sexual partners within the past year.

BC, Black Caribbean; SHC, sexual health clinic; WBI, White British/Irish.

BC and WBI women were similar in age (medians: 26 and 25 years, respectively). Fewer BC women than WBI women were educated beyond GCSE equivalent, but they were equally likely to be employed. No ethnic differences were observed in women’s current partnership status: over half reported only committed partnership(s); under a third reported only casual partnership(s); and less than 2% both committed and casual partnerships. BC women reported fewer sexual partners than WBI women, for example, 51.0% of BC women reported just one partner in the past year compared with 42.3% of WBI women; 7.2% of BC women reported ≥5 partners in this time frame, compared with 15.4% of WBI women. BC women also reported fewer *new* partners, and were three times as likely to report no new partners (14.6%; WBI women: 4.8%). Among women reporting 2+ partners (past year), around 40% reported concurrency, with no ethnic difference.

We now consider gender differences, focusing on BC attendees (the population of interest). BC men and women were similar in age, and while BC women were more likely to be educated beyond GCSEs, they were equally likely to be employed. BC women were more likely than BC men to report only current committed partnership(s) (52.3% vs 33.5%), and less likely to report only current casual partnership(s) (31.1% vs 41.5%). In the past year, BC women reported fewer partners, and fewer new partners, than BC men (eg, 51.0% of BC women, but 16.1% of BC men, reported only one partner). Among those reporting 2+ partners (past year), BC women were less likely than BC men to report concurrency (41.7% vs 57.2%).

### Most recent opposite-sex partnerships

We now present the analyses (table 2) of the partnership-level data set.

**Table 2:** Characteristics of participants’ most recent opposite-sex partnerships in the past 3 months, by ethnic group and gender

	Men’s partnerships			Women’s partnerships			P for gender differences	
	BC men’s partnerships	WBI men’s partnerships	P for ethnic difference	BC women’s partnerships	WBI women’s partnerships	P for ethnic difference	Among BC attendees’ partnerships	Among WBI attendees’ partnerships
Denominator (number of partnerships reported)*	*324*	*689*		*449*	*1041*			
**Partnership type** at most recent sex with the partner—5 categories			0.001			0.028	<0.001	<0.001
Married	2.2%	3.5%		3.2%	3.4%			
Committed relationship but not married	23.9%	22.7%		45.8%	39.4%			
Regular partners but not in a committed relationship	35.4%	20.7%		32.3%	26.2%			
Had recently met	28.3%	32.6%		15.6%	23.0%			
Had just met	10.2%	20.5%		3.2%	8.0%			
**Partnership type** at most recent sex with the partner—3 categories¶			<0.001			<0.001	<0.001	<0.001
Committed (married +committed but unmarried)	*82*	*179*		*217*	*444*			
	26.1%	26.2%		49.0%	42.8%			
Uncommitted regular (regular partners but not in a committed relationship)	*111*	*141*		*143*	*272*			
	35.4%	20.7%		32.3%	26.2%			
Casual (recently met+just met)	*121*	*362*		*83*	*321*			
	38.5%	53.1%		18.7%	31.0%			
**Partnership duration (at most recent sex)** **†**			0.112			<0.001	<0.001	<0.001
<4 weeks	35.1%	35.2%		23.1%	24.1%			
1–6 months	36.5%	42.9%		29.1%	39.8%			
>6 months	28.4%	21.9%		47.8%	36.2%			
*By partnership type at most recent sex*								
*Committed*			0.085			0.098	0.190	0.546
<4 weeks	18.7%	8.4%		10.2%	6.9%			
1–6 months	29.3%	28.6%		19.4%	27.5%			
>6 months	52.0%	63.0%		70.4%	65.6%			
*Uncommitted regular*			0.125			0.151	0.728	0.250
<4 weeks	21.5%	17.4%		18.4%	11.4%			
1–6 months	40.9%	56.0%		40.8%	57.9%			
>6 months	37.6%	26.6%		40.8%	30.7%			
*Casual*			0.247			0.397	0.685	0.306
<4 weeks	58.5%	54.1%		64.5%	56.4%			
1–6 months	38.7%	45.0%		34.2%	41.3%			
>6 months	2.8%	0.9%		1.3%	2.3%			
**Expectation of sex again: Yes/probably** **‡**	58.3%	46.2%	<0.001	69.1%	60.5%	0.004	0.016	<0.001
*By partnership type at most recent sex*								
Committed	79.0%	85.5%	0.276	82.2%	84.0%	0.615	0.522	0.614
Uncommitted regular	65.0%	62.0%	0.566	65.4%	64.6%	0.745	0.937	0.372
Casual	36.9%	21.4%	0.007	43.2%	23.9%	0.003	0.115	0.319
**Non-use of condom at most recent sex**	58.7%	64.2%	0.158	67.5%	68.6%	0.758	0.088	0.207
*By partnership type at most recent sex*								
Committed	61.3%	71.8%	0.091	74.8%	76.9%	0.512	0.034	0.033
Uncommitted regular	62.4%	65.2%	0.677	64.8%	67.0%	0.686	0.655	0.726
Casual	54.7%	60.7%	0.321	55.7%	58.7%	0.584	0.892	0.722
**How they met the partner** **§**			0.427			0.005	0.070	0.003
School/college/university/work	23.5%	24.0%		23.5%	30.0%			
Social venue/public place/neighbour	27.6%	30.6%		24.4%	20.3%			
Through friends/family	21.9%	17.4%		30.0%	23.7%			
Online, including internet dating	18.2%	16.8%		12.2%	19.1%			
Other	8.8%	11.1%		9.9%	6.8%			

*Denominators: partnerships reported by participants (SHC attendees) identifying as male, and who reported only female partners in the past year; and partnerships reported by participants identifying as female who reported only male partners in the past year. For categorical outcome variables, Pearson χ^2^ tests were used to calculate p values.

†Estimated from survey questions about recency of first, and of most recent sex.

‡Alternative responses included ‘I don’t know’ as well as ‘no’ and ‘probably not’.

§Categories in the table are based on a larger number of response options, as follows: school/college/university/work: ‘At school’, ‘At college/university’, ‘At work (or through work)’; social venue/public place/neighbour: ‘In a pub, bar, night club, disco, or dance’, ‘Through a sports club, faith group, or other organisation’, ‘Neighbour/lived locally/flat share’, ‘In a public place (eg, park, café, shop, public transport)’; through friends/family: ‘Introduced by friends or family’, ‘Had always known each other (eg, as family friends)’, ‘Arranged marriage’; online, including internet dating: ‘Internet dating website’, ‘Facebook’, ‘Twitter’, ‘Instagram’, ‘Pandora’, ‘WhatsApp’, ‘Other social media websites’, ‘Online but not through dating website or social media’; other: ‘On holiday or while travelling’, ‘Other dating agency/personal ads’, ‘Partner was a sex worker’, ‘Partner was my client’, ‘Other’.

¶Numbers in each category are italicised.

BC, Black Caribbean; SHC, sexual health clinic; WBI, White British/Irish.

At most recent sex, BC and WBI men’s reported partnerships were equally likely, at around one-quarter, to be committed, however 35.4% of BC men’s partnerships were uncommitted regular, compared with 20.7% of WBI men’s partnerships. Casual partnerships comprised 38.5% of BC men’s partnerships, but 53.1% of WBI men’s partnerships. Despite these differences, we observed no ethnic differences in partnership duration to date (estimated from dates of first and most recent sex): around 35% of men’s partnerships had lasted under 4 weeks, close to 40% had lasted 1–6 months, and around one-quarter were longer still. We also observed no ethnic differences in durations of different partnership types (unsurprisingly, committed partnerships tended to be longest, and casual partnerships shortest). A higher proportion of BC men’s *casual* partnerships were expected to be ongoing, compared with WBI men’s casual partnerships (58.3% vs 46.2%) with no ethnic difference in this expectation among other partnership types. Reported non-use of condoms at last sex (around 60%) was similar between BC and WBI men’s partnerships. Men’s partners were most commonly met through social venues (approximately 30%), one-quarter were met through education or employment and around 17% online, with no ethnic differences.

Compared with WBI women’s partnerships, BC women’s reported partnership type at most recent sex was more often committed (49.0% vs 42.8%) or uncommitted regular (32.3% vs 26.2%), and less commonly casual (18.7% vs 31.0%). Less than one-quarter of BC and WBI women’s partnerships had an estimated duration of less than 4 weeks, but almost half (47.8%) of BC women’s partnerships had lasted longer than 6 months, compared with 36.2% of WBI women’s partnerships. As with men’s partnerships, BC women’s *casual* partnerships were more likely than those of WBI women to be ongoing (69.1% vs 60.5%), with no ethnic differences for other partnership types. Non-use of condoms at last sex was reported in over two-thirds of women’s partnerships, with no ethnic difference. We observed differences in where women’s partners were met, for example, BC women’s partners were most commonly met through friends/family (30.0%), compared with 23.7% of WBI women’s partners; 12.2% of BC women’s partners were met online, compared with 19.1% of WBI women’s partners.

Compared with BC men’s partnerships, BC women’s partnerships were more often committed and less often casual, tended to be longer and were more likely expected to be ongoing. No statistically significant gender differences were observed in condom use at last sex, nor in where BC men’s and women’s partners were met.

### Age/ethnic-mixing in most recent opposite-sex partnerships

BC men were typically a few years older than their partners (table 3). The majority of their partnerships involved ethnic-mixing (67.3%), and among committed partnerships, over a third involved age-mixing (35.9%). Ethnic-mixing (35.1%) and age-mixing (23.1%) were considerably less common in WBI men’s partnerships.

**Table 3:** Age/ethnic-mixing in opposite-sex partnerships in the past 3 months, by ethnic group and gender

	Men’s partnerships			Women’s partnerships			P for gender differences	
	BC men’s partnerships	WBI men’s partnerships	P for ethnic difference	BC women’s partnerships	WBI women’s partnerships	P for ethnic difference	Among BC attendees’ partnerships	Among WBI attendees’ partnerships
*Denominator (number of partnerships reported)* *	*324*	*689*		*449*	*1041*			
**Age-mixing**								
Median age difference (IQR) (man’s age minus woman’s age)†	2 (0–4)	1 (−1 to 4)		2 (0–4)	1 (0–4)			
% of partnerships with age-mixing (≥5 years age difference)	29.2%	29.0%	0.967	31.6%	25.5%	0.013	0.614	0.212
*By partnership type at most recent sex*								
Committed	35.9%	23.1%	0.040	32.7%	23.8%	0.022	0.572	0.869
Uncommitted regular	31.4%	30.3%	0.836	26.7%	25.3%	0.703	0.343	0.205
Casual	21.8%	31.5%	0.186	36.5%	27.8%	0.186	0.058	0.380
% of partnerships with man ≥5 years older than woman	24.2%	21.7%	0.515	25.7%	20.4%	0.097	0.744	0.641
*By partnership type at most recent sex*								
Committed	29.5%	17.8%	0.038	26.9%	20.3%	0.111	0.602	0.474
Uncommitted regular	25.5%	22.7%	0.498	22.2%	19.2%	0.427	0.439	0.603
Casual	18.2%	23.4%	0.416	27.0%	21.9%	0.361	0.215	0.681
**Ethnic-mixing**								
% of partnerships with ethnic-mixing‡	67.3%	35.1%	<0.001	39.6%	32.1%	0.231	<0.001	0.462
*By partnership type at most recent sex*								
Committed	57.7%	26.4%	<0.001	33.5%	26.5%	0.303	0.001	0.986
Uncommitted regular	64.2%	37.7%	<0.001	36.8%	35.5%	0.811	<0.001	0.684
Casual	78.0%	38.9%	<0.001	58.5%	37.5%	0.009	0.011	0.777
Partner’s ethnic group			<0.001			<0.001	<0.001	0.018
White British/Irish	26.7%	64.9%		7.4%	67.9%			
White other	5.7%	18.5%		1.6%	10.4%			
Black African	8.3%	2.1%		18.5%	4.6%			
Black Caribbean	32.7%	2.4%		60.4%	7.0%			
Indian/Pakistani/Bangladeshi	3.7%	2.0%		0.7%	2.2%			
Mixed	15.7%	2.4%		7.2%	5.0%			
Other Asian/Chinese/Arab/other	7.3%	7.8%		4.2%	3.0%			

*Denominators: partnerships reported by participants (SHC attendees) identifying as male, and who reported only female partners in the past year; and partnerships reported by participants identifying as female who reported only male partners in the past year. Pearson χ^2^ tests were used to calculate p values.

†If a positive value, the man is older than the woman, and if a negative value, the woman is older than the man.

‡Defined as partners of different ethnic groups to the participant.

BC, Black Caribbean; SHC, sexual health clinic; WBI, White British/Irish.

BC women were typically a few years younger than their partners, and WBI women slightly closer in age. Almost one-third of BC women’s partnerships involved age-mixing (31.6%), and over one-third involved ethnic-mixing (39.6%). By comparison, WBI women’s partnerships less commonly involved ethnic-mixing (25.5%), but ethnic-mixing was similarly common, except in casual partnerships in which ethnic-mixing was more common among BC women’s than WBI women’s partnerships (58.5% vs 37.5%).

No gender differences were observed in the proportions of partnerships involving age-mixing. BC men’s partners were more likely than BC women’s partners to be of non-BC ethnicity.

### The role of sexual mixing and partnership type in explaining ethnic and gender differences in acute STI diagnosis

Relative to WBI men, BC men had elevated odds of diagnosis with a bacterial STI/trichomoniasis around the time of survey completion in both the crude and adjusted analyses (OR range: 1.36–1.53), but the 95% CIs all overlap one, meaning that these may also indicate lower odds or no difference between the ethnic groups (table 4). Contrastingly, acute STIs were more common among BC women than WBI women (16.0% vs 7.7%, OR: 2.29, 95% CI 1.23 to 4.27). In both ethnic groups, men were more likely than women to have acute STI. Effect sizes hardly changed after adjusting for age and numbers of recent partners (potential confounders), or after additionally adjusting for sexual mixing variables and recent partnership type(s).

**Table 4:** The roles of sexual mixing and partnership type in explaining ethnic and gender differences in acute STI diagnosis

	Men				Women				Comparisons by gender			
	BC % (95% CI)	WBI % (95% CI)	OR: BC compared with WBI (95% CI)	P value	BC %	WBI %	OR: BC compared with WBI (95% CI)	P value	Among BC attendees		Among WBI attendees	
									OR: men compared with women (95% CI)	P value	OR: men compared with women (95% CI)	P value
*Denominator (participants)* *	*149*	*364*			*319*	*664*						
‘Acute STI’: Bacterial STI and/or trichomoniasis diagnosis/es† within ±6 weeks of clinic attendance (95% CI)	26.8% (15.2% to 42.9%)	20.1% (15.9% to 25.0%)			16.0% (9.9%–24.7%)	7.7% (5.4%–10.9%)						
Unadjusted OR (95% CI)			1.46 (0.69 to 3.10)	0.294			2.29 (1.23 to 4.27)	0.014	1.93 (1.26 to 2.96)	0.006	3.02 (1.95 to 4.68)	<0.001
aOR1: adjusted for age and number of recent partners‡ (95% CI)			1.36 (0.63 to 2.93)	0.402			2.31 (1.28 to 4.14)	0.009	1.82 (1.14 to 2.92)	0.017	2.76 (1.81 to 4.21)	<0.001
aOR2: adjusted for the above variables, and sexual mixing and partnership type§ (95% CI)			1.53 (0.57 to 4.06)	0.367			2.15 (1.06 to 4.38)	0.037	2.01 (1.21 to 3.33)	0.011	2.70 (1.61 to 4.51)	<0.001

*Denominators: participants (SHC attendees) identifying as male, and who reported only female partners in the past year; and participants identifying as female who reported only male partners in the past year.

†The following bacterial STIs: chlamydia, gonorrhoea, syphilis, chancroid, lymphogranuloma venereum (LGV), non-specific genital infection (NSGI), *Mycoplasma genitalium* infection, shigellosis, non-specific pelvic inflammatory disease (PID), donovanosis (no one in the sample was diagnosed with the latter); and also trichomoniasis, caused by the flagellated protozoan parasite *Trichomonas vaginalis*.

‡Adjusted for: age as a continuous variable, and number of opposite-sex partners in the past 3 months (0, 1, 2+).

§Adjusted for: age as a continuous variable, and number of opposite-sex partners in the past 3 months (0, 1, 2+), and the following (all derived from questions about the (up to) three most recent partners within the past 3 months): any committed partners within the past 3 months, any uncommitted regular partners within the past 3 months, any casual partners within the past 3 months; and the following sexual mixing variables: any age-mixing* among partners within the past 3 months, any ethnic-mixing among partners within the past 3 months. (*We repeated this analysis replacing ‘any age-mixing’ with a variable for any age-mixing in which the man was ≥5 years older. Results were very similar, as shown in online supplementary table 5.)

BC, Black Caribbean; SHC, sexual health clinic; WBI, White British/Irish; aOR, adjusted OR.

## Discussion

### Main findings

In this large study of behaviourally-heterosexual people attending SHCs across England, we found ethnic differences in partnership types. Compared with those of WBI attendees, BC attendees’ partnerships were more commonly ‘uncommitted regular’, less commonly ‘casual’ and were more likely to involve age/ethnic-mixing. We also found gender differences by partnership type, with BC women’s partnerships more often committed than BC men’s. Taking account of ethnic differences in partnership characteristics did not explain the greater likelihood of acute STI observed among women of BC ethnicity in our study. Despite our attempts to go beyond the individual-level perspective, we found little evidence that partnership characteristics explain the differences in STI diagnosis in the population.

### Strengths and weaknesses of the study

Our descriptive data on SHC attendees of BC ethnicity (who are at elevated STI risk)^24 25^ use more detailed measures than currently available from routine STI surveillance. By purposively selecting clinics, we attained a large sample of this epidemiologically important population, compared with clinic surveys targeting Black/BC attendees.^24 31^ Nonetheless, statistical power issues led us to adjust only for age and partner numbers (known confounders),^25^ alongside partnership-level and mixing variables (this paper’s focus), and may have limited our ability to detect differences by partnership type. Ethnic differences in employment (men only) and education perhaps indicate that these variables, and deprivation, could be influential, but large differences in STI diagnosis rates have been shown to remain after adjusting for deprivation.^9^ Use of statistical significance to inform variable inclusion may have excluded important factors for which we had data, aside from factors for which we did not.

Our findings are somewhat more difficult to interpret than those of nationally representative surveys,^23 25^ because ethnic and gender differences may be diluted among the high-risk population of SHC attendees.^25 26^ However, we used a clinic-verified outcome, in conjunction with detailed patient-reported data. Response and linkage rates were relatively high for a clinic survey (see online supplementary figure 1).^28^ Unlike many SHC surveys, we captured data on attendees’ three most recent partnerships, of relevance to acute STI and minimising recall bias. However, participants’ reports of their partners’ ages and ethnicities may be unreliable, especially for casual partners. Our measure of condom use at most recent sex is an indicator of capacity for risk reduction, but may poorly reflect STI risk because individuals may not use condoms regularly, or at all, with their steady partners.^32^ Furthermore, interethnic differences in condom use could contribute to observed differences in acute STI. We used standard ethnicity categories,^13^ but these may conceal considerable within-group heterogeneity, and our definition of ethnic-mixing may not match public understandings of ‘mixed-race’/‘interracial’ partnerships.

10.1136/sextrans-2018-053739.supp2 Supplementary data

Our qualitative research^33^ informed the need for an ‘uncommitted regular’ partnership category, enabling us to transcend the regular/casual dichotomy. Participants’ selection of predefined labels to describe partnership type(s) was likely influenced by sociocultural and gender norms that may vary within our sample,^16 34^ although social desirability effects were likely minimised by the online survey mode.^28^ We could not explore participants’ partners’ perceptions of partnerships, nor partners’ sexual behaviour, which could have helped in understanding partnerships’ STI transmission risk.

### Discussion of findings in relation to other studies

Our study among SHC attendees observed a greater likelihood of acute STI diagnosis among BC women compared with WBI women, and while the same pattern was observed among men, it did not reach statistical significance. Men’s attendances may be more likely prompted by symptoms predictive of an STI, as men in both ethnic groups were more commonly diagnosed than women. This pattern mirrors self-reported STI diagnosis data from the general population in Britain^3^ and other UK studies among SHC attendees (eg, Coyle *et al* 31) despite methodological differences in ethnicity categories, population, time frame and STIs studied.^9^ Ethnic differences in attendees’ reported (hetero)sexual risk behaviours and partnership characteristics were not patterned as would be expected given disproportionately high STI diagnosis rates in the BC population^9 35^ particularly among the BC women in this study. Other studies have also found mismatches between behaviour and STI risk,^36 37^ and specifically that BC women report lower or similar levels of sexual risk behaviour, and BC men have similar or higher reported risk behaviours than other ethnic groups, but both BC men and BC women have higher levels of diagnosed STI.^31^ This highlights the likely influence of partnership and network characteristics in STI acquisition risk.^37 38^ Although our study goes beyond other descriptive studies by examining ethnic differences in partnership characteristics in greater detail, it is perhaps unsurprising that our cross-sectional study does not ‘explain’ findings that result from non-linear transmission dynamics, because such complex relationships may be oversimplified by linear statistical models. With these non-linear dynamics, the effects of small differences in behaviour, especially in small populations, may be amplified.^39 40^ However, mathematical modelling studies which dynamically model the spread of infection through sexual networks may reconcile these findings.^21 41^

In partnership-level analyses, we found higher ethnic-mixing than is reported in the general population,^18^ which varied by attendees’ ethnic group and partnership type. This may reflect the higher prevalence of uncommitted/casual partnerships (which are more commonly disassortative) among SHC attendees, and recruitment from clinics serving ethnically diverse populations. We confirmed others’ findings that uncommitted regular partnerships and concurrency (for men) are relatively common in the BC population,^14 15 33^ but these were prevalent throughout our SHC attending sample.

In a separate analysis of BBEST data (conducted among participants of all sexualities), we found few ethnic differences in reasons for SHC attendance, for instance, there were no statistically significant ethnic differences in the proportions of men and (separately) women reporting attending because of experiencing symptoms, or because they wanted an asymptomatic check-up.^42^ Compared with their WBI counterparts, BC women’s attendance was more commonly related to recent bacterial STI diagnoses, and BC men’s to their *partners’* symptoms or STI diagnosis, which reflects differences in STI risk.

### Meaning and implications

We found little evidence that partnership and mixing characteristics explain ethnic differences in SHC attendees’ likelihood of STI diagnosis. However, this may reflect in part *how* we characterised partnerships and does not necessarily mean that attempts to account for the partnership-level perspective are unwarranted. Rather, there is a need for more sophisticated measures—a challenge when data collection currently occurs at the individual level — ideally going beyond the individual (index) patient to take account of their partners’ behaviour and characteristics. Developing a deeper understanding of broader sexual network characteristics may help explain the greater STI risk in the BC population.^37^ As a first step, there is a need to better characterise ‘high transmission networks’ for bacterial STIs and trichomoniasis within the BC population (ie, sexual networks among which infection transmission is higher than in the BC population as a whole). This could be done through sexual network analysis, and also by accounting for partnership type,^36^ especially if interventions developed to reduce STI transmission, prevalence and sexual health inequalities take a multilevel approach. Our study has begun this process. For example, we found that BC men’s sexual networks may tend to be more ‘open’, and BC women’s more ‘closed’, which could amplify BC women’s STI risk.

Our findings challenge individualised explanations of STI risk, particularly for BC women SHC attendees. Furthermore, clinicians interpreting sexual histories, and those designing and delivering health promotion interventions, should not assume that the sociosexual/partnership norms of the ethnic majority are universal. Our distinction within conventionally termed ‘casual’ partnerships may be particularly important for prioritising partner notification, as many ‘uncommitted regular’ partnerships are likely ongoing, with implications for reinfection if such partners are untreated.

### Unanswered questions and future research

Sexual network studies, informed by qualitative and ethnographic work and more refined partnership-type studies, could inform the characterisation of high transmission networks for bacterial STIs and trichomoniasis in the BC population. Ethnic differences in (hetero)sexual practices, and how these may differ by partnership type, require further exploration.

**Key messages**

- We investigated whether partnership and sexual mixing characteristics could explain ethnic inequalities in STI, focusing on people of Black Caribbean ethnicity attending sexual health clinics.
- We found people of Black Caribbean ethnicity differed in their reporting of sexual mixing and partnerships, compared with White British/Irish clinic attendees, but these differences did not explain differences in acute STI diagnoses.
- Further studies are needed which investigate the sexual networks of populations at elevated risk of STIs, in order to inform appropriate interventions.
