# Supplementary material for: Ethnic variations in sexual partnerships and mixing, and their association with STI diagnosis: findings from a cross-sectional biobehavioural survey of attendees of sexual health clinics across England
Source: Sex Transm Infect. 2019 Aug 17;96(4):283–92. doi: 10.1136/sextrans-2018-053739 (PMC7279208; doi:10.1136/sextrans-2018-053739)
Supplement: Supplementary data [file sextrans-2018-053739supp001.pdf]

Web appendices: Supplementary Tables 1-5

We present results for a larger range of ethnic groups (than BC and WBI). We excluded from these tables 208 men and women participants of Chinese, ‘other Asian’, Arab, and ‘other ethnicity’ groups, because the numbers of participants in each group were small (n=46, n=55, n=33, n=74, respectively), and treating them as one heterogeneous group would not be particularly meaningful (matching a previously-published approach[4]). We also excluded these people’s reported partnerships.

Forty people in our sample selected ‘Black Other’ as their ethnic group, the largest proportion of which reported two different ethnic groups for their parents, so for the purposes of our study, we re-coded all Black Other participants to Mixed ethnicity.

Supplementary Table 1A: Variations in the prevalence of reported number and types of sexual partners by ethnic group: Men

	**White British/ Irish**	**White other**	**Black African**	**Black Caribbean**	**Indian/ Pakistani/ Bangladeshi**	**Mixed ethnicity**	**All [2]**	p for ethnic difference
Denominator (participants)[1]	*426*	*134*	*152*	*182*	*89*	*101*	*1084*	
**Sociodemographics**								
Age (median, IQR)	27 (24-31)	28 (25-33)	27 (23-36)	27 (22-33)	28 (25-33)	26 (22-30)	27 (23-32)	
								0.027
15-19	4.9%	3.7%	5.3%	11.0%	5.6%	8.9%	6.3%	
20-24	23.9%	20.1%	27.6%	25.8%	19.1%	35.6%	25.0%	
25-34	54.2%	56.7%	36.2%	42.3%	53.9%	47.5%	49.4%	
35-44	12.0%	14.2%	21.7%	11.0%	19.1%	5.9%	13.5%	
45+	4.9%	5.2%	9.2%	9.9%	2.2%	2.0%	5.9%	
Education above GCSEs, or equivalent[3]	82.2%	87.9%	78.9%	65.9%	88.6%	78.2%	79.9%	0.004
In employment	84.0%	85.7%	75.5%	71.7%	73.6%	75.0%	79.3%	0.002
**Current partnership(s) status**								0.140
Committed sexual partnership(s) only	38.6%	41.%	43.2%	33.5%	43.2%	34.0%	38.7%	

Casual sexual partnership(s) only	38.6%	35.1%	37.2%	41.5%	26.1%	44.0%	37.9%	
Both committed and casual sexual partnerships	2.6%	3.7%	4.1%	7.4%	9.1%	5.0%	4.5%	
No current sexual partnership	20.1%	20.1%	15.5%	17.6%	21.6%	17.0%	18.9%	
**Sexual partners, past year**								
Number of partners								0.063
1	25.4%	26.6%	23.8%	16.1%	38.3%	23.5%	24.6%	
2	15.9%	13.3%	21.8%	23.0%	14.8%	12.2%	17.1%	
3-4	26.1%	25.0%	20.4%	23.6%	27.2%	28.6%	25.0%	
5-9	21.2%	22.7%	24.5%	24.7%	12.3%	14.3%	21.1%	
10+	11.5%	12.5%	9.5%	12.6%	7.4%	21.4%	12.1%	
Number of *new* partners								0.031
0	3.2%	3.2%	9.7%	8.9%	8.4%	10.3%	6.1%	
1	36.7%	34.4%	40.3%	34.9%	43.4%	33.0%	36.8%	
2+	60.1%	62.4%	50.0%	56.2%	48.2%	56.7%	57.1%	
Overlapping (concurrent) partnerships, *among those reporting two or more partners in the past year*[4]								0.018
Denominator (participants):	*310*	*94*	*113*	*145*	*51*	*75*	*788*	
no	43.5%	42.6%	31.0%	33.1%	43.1%	30.7%	38.5%	
yes	50.6%	50.0%	54.0%	57.2%	45.1%	56.0%	52.4%	
don't remember	5.8%	7.4%	15.0%	9.7%	11.8%	13.3%	9.1%	
**Sexual partners, past 3 months**								0.050
0	8.8%	10.6%	4.0%	7.3%	16.1%	10.1%	8.8%	
1	43.4%	46.2%	46.4%	32.6%	48.3%	35.4%	42.0%	
2	23.6%	22.7%	23.2%	28.1%	20.7%	25.3%	24.1%	
3	12.9%	8.3%	9.3%	15.2%	8.0%	9.1%	11.4%	
4+	11.2%	12.1%	17.2%	16.9%	6.9%	20.2%	13.6%	

Notes for Supplementary Table 1A: [1]Denominators: participants (SHC attendees) identifying as male, and who reported only female partners in the past year.
[2]Excludes Other Asian/Chinese/Arab/Other.
[3]GCSE: General Certificate of Secondary Education, exams typically taken by age 16 in England.
[4]From a direct question about overlapping partnerships.

Supplementary Table 1B: Variations in the prevalence of reported number and types of sexual partners and ethnic group: Women

	**White British/ Irish**	**White other**	**Black African**	**Black Caribbean**	**Indian/ Pakistani/ Bangladeshi**	**Mixed ethnicity**	**All [2]**	p for ethnic difference
Denominator (participants)[1]	*792*	*314*	*236*	*390*	*149*	*265*	*2146*	
**Sociodemographics**								
Age (median, IQR)	25 (21-30)	27 (23-32)	25 (21-29)	26 (22-31)	24 (21-30)	24 (20-28)	25 (21-30)	
								0.010
15-19	10.1%	5.7%	14.8%	12.8%	13.4%	19.6%	11.9%	
20-24	36.9%	27.1%	34.7%	32.8%	38.3%	32.8%	34.1%	
25-34	40.5%	49.4%	36.4%	37.4%	36.2%	40.0%	40.4%	
35-44	8.3%	15.0%	10.6%	10.3%	11.4%	6.4%	9.9%	
45+	4.2%	2.9%	3.4%	6.7%	0.7%	1.1%	3.7%	
Education above GCSEs, or equivalent[3]	84.8%	84.1%	82.8%	78.8%	89.9%	76.7%	82.8%	0.003
In employment	71.3%	79.0%	62.6%	71.1%	63.5%	67.4%	70.4%	0.025
**Current partnership(s) status**								0.157
Committed sexual partnership(s) only	50.1%	51.3%	54.9%	52.3%	61.2%	53.6%	52.4%	
Casual sexual partnership(s) only	28.9%	27.9%	24.5%	31.1%	19.7%	24.9%	27.6%	
Both committed and casual sexual partnerships	1.8%	3.2%	0.9%	1.8%	3.4%	4.6%	2.4%	
No current sexual partnership	19.2%	17.6%	19.7%	14.8%	15.6%	16.9%	17.7%	
**Sexual partners, past year**								
Number of partners								0.005
1	42.3%	41.8%	54.5%	51.0%	54.2%	45.3%	46.3%	
2	19.3%	19.2%	27.0%	24.2%	22.5%	22.8%	21.7%	
3-4	23.0%	20.9%	14.0%	17.6%	14.1%	18.1%	19.5%	
5-9	12.6%	15.8%	3.2%	6.1%	6.3%	10.6%	10.2%	

10+	2.8%	2.4%	1.4%	1.1%	2.8%	3.1%	2.3%	
Number of new partners								<0.001
0	4.8%	6.5%	8.9%	14.6%	5.5%	6.7%	7.6%	
1	53.7%	50.5%	69.3%	64.9%	69.0%	62.1%	59.0%	
2+	41.5%	43.0%	21.8%	20.5%	25.5%	31.2%	33.4%	
Overlapping (concurrent) partnerships, *among those reporting two or more partners in the past year*[4]								0.046
Denominator (participants):	*453*	*180*	*107*	*192*	*68*	*145*	*1145*	
no	58.7%	48.9%	57.0%	55.2%	51.5%	57.2%	55.8%	
yes	39.7%	44.4%	34.6%	41.7%	45.6%	37.9%	40.4%	
don't remember	1.5%	6.7%	8.4%	3.1%	2.9%	4.8%	3.8%	
**Sexual partners, past 3 months**								0.012
0	6.9%	5.5%	13.4%	11.3%	5.6%	8.5%	8.3%	
1	63.6%	67.2%	70.5%	66.5%	73.4%	66.0%	66.3%	
2	17.8%	17.4%	11.2%	15.0%	11.2%	15.8%	15.8%	
3	5.5%	6.1%	4.5%	4.0%	5.6%	5.0%	5.1%	
4+	6.3%	3.9%	0.4%	3.2%	4.2%	4.6%	4.4%	

Notes for Supplementary Table 1B: [1]Denominators: participants (SHC attendees) identifying as female, and who reported only male partners in the past year.

[2]Excludes Other Asian/Chinese/Arab/Other.

[3]GCSE: General Certificate of Secondary Education, exams typically taken by age 16 in England.

[4]From a direct question about overlapping partnerships.

Supplementary Table 2A: Characteristics of male participants' most recent opposite-sex partnerships in the past 3 months, by ethnic group

	White British/ Irish	White other	Black African	Black Caribbean	Indian/ Pakistani/ Bangladeshi	Mixed ethnicity	All[2]	p for ethnic differ-ence
Denominator (number of partnerships reported)[1]	*682*	*196*	*244*	*314*	*117*	*170*	*1723*	
**Partnership type** at most recent sex with the partner								0.001
Married	3.5%	6.1%	4.9%	2.2%	11.1%	1.8%	4.1%	
Committed relationship but not married	22.7%	21.9%	26.2%	23.9%	13.7%	22.9%	22.8%	
Regular partners (but not in a committed relationship)	20.7%	24.0%	31.1%	35.4%	21.4%	27.6%	25.9%	
Had recently met	32.6%	30.1%	25.4%	28.3%	22.2%	30.0%	29.5%	
Had just met	20.5%	17.9%	12.3%	10.2%	31.6%	17.6%	17.6%	
**Partnership type** at most recent sex with the partner – 3 categories								0.005
Committed (Married+committed but unmarried)	26.2%	28.1%	31.1%	26.1%	24.8%	24.7%	26.9%	
	179	*55*	*76*	*82*	*29*	*42*	*463*	
Uncommitted but regular (Regular partners but not in a committed relationship)	20.7%	24.0%	31.1%	35.4%	21.4%	27.6%	25.9%	
	141	*47*	*76*	*111*	*25*	*47*	*447*	
Casual (Recently met+just met)	53.1%	48.0%	37.7%	38.5%	53.8%	47.6%	47.2%	
	362	*94*	*92*	*121*	*63*	*81*	*813*	
**Partnership duration (at most recent sex)**[3]								0.114
< 4 weeks	35.2%	32.0%	33.2%	35.1%	28.2%	44.1%	34.9%	
1-6 months	42.9%	50.0%	38.4%	36.5%	44.5%	35.2%	41.3%	
>6 months	21.9%	18.0%	28.4%	28.4%	27.3%	20.7%	23.8%	
By partnership type at most recent sex:								
Committed								
< 4 weeks	8.4%	10.4%	19.7%	18.7%	7.4%	16.2%	13.0%	0.185

1-6 months	28.6%	41.7%	28.8%	29.3%	33.3%	32.4%	31.0%	
>6 months	63.0%	47.9%	51.5%	52.0%	59.3%	51.4%	56.0%	
Uncommitted but regular								
< 4 weeks	17.4%	21.1%	21.2%	21.5%	18.2%	24.2%	20.2%	0.249
1-6 months	56.0%	65.8%	48.5%	40.9%	31.8%	45.5%	49.3%	
>6 months	26.6%	13.2%	30.3%	37.6%	50.0%	30.3%	30.5%	
Casual								
< 4 weeks	54.1%	50.0%	54.1%	58.5%	41.4%	67.1%	54.5%	0.076
1-6 months	45.0%	47.7%	37.8%	38.7%	56.9%	32.9%	43.4%	
>6 months	0.9%	2.3%	8.1%	2.8%	1.7%	0.0%	2.1%	
**Expectation of sex again: Yes/probably[4]**	46.2%	55.0%	54.8%	58.3%	46.2%	53.1%	51.3%	0.008
By partnership type at most recent sex:								
Committed	85.5%	88.9%	78.7%	79.0%	92.9%	77.5%	83.3%	0.270
Uncommitted but regular	62.0%	56.5%	65.7%	65.0%	64.0%	75.0%	64.2%	0.354
Casual	21.4%	33.3%	28.1%	36.9%	16.1%	28.0%	26.0%	0.015
**Non-use of condom at most recent sex**	64.2%	53.3%	59.6%	58.7%	46.6%	62.9%	59.9%	0.009
By partnership type at most recent sex:								
Committed	71.8%	70.9%	71.1%	61.3%	58.6%	64.3%	68.2%	0.276
Uncommitted but regular	65.2%	59.6%	61.3%	62.4%	64.0%	71.7%	63.9%	0.648
Casual	60.7%	40.9%	47.8%	54.7%	32.2%	56.4%	53.4%	0.012
**How met partner[5]**								0.049
school/college/university/work	24.0%	20.8%	27.0%	23.5%	23.3%	27.3%	24.3%	
social venue	30.6%	32.5%	31.0%	27.6%	23.3%	36.0%	30.4%	
through friends/family	17.4%	17.8%	23.4%	21.9%	15.0%	9.9%	18.2%	
online including internet dating	16.8%	21.8%	12.1%	18.2%	14.2%	13.4%	16.4%	
Other	11.1%	7.1%	6.5%	8.8%	24.2%	13.4%	10.7%	

Notes for Supplementary Table 2A: [1]Denominators: partnerships reported by participants (SHC attendees) identifying as male, and who reported only female partners in the past year.

[2]Excludes Other Asian/Chinese/Arab/Other.
[3]Estimated from questions about recency of first, and of most recent sex
[4]Alternative responses included ‘I don’t know’ as well as ‘no’ and ‘probably not’.
[5]Categories in the table are based on a larger number of response options, as follows: school/college/university/work: ‘At school’, ‘At college/university’, ‘At work (or through work)’; social venue/public place/neighbour: ‘In a pub, bar, night club, disco, or dance’, ‘Through a sports club, faith group, or other organisation’, ‘Neighbour/lived locally/flat share’, ‘In a public place (e.g. park, café, shop, public transport)’; through friends/family: ‘Introduced by friends or family’, ‘Had always known each other (e.g. as family friends)’, ‘Arranged marriage’; online, including internet dating: ‘Internet dating website’, ‘Facebook’, ‘Twitter’, ‘Instagram’, ‘Pandora’, ‘Whatsapp’, ‘Other social media websites’, ‘Online but not through dating website or social media’; other: ‘On holiday or while travelling’, ‘Other dating agency/personal ads’, ‘Partner was a sex worker’, ‘Partner was my client’, ‘Other’.

Supplementary Table 2B: Characteristics of female participants' most recent opposite-sex partnerships in the past 3 months, by ethnic group

	**White British/Irish**	**White other**	**Black African**	**Black Caribbean**	**Indian/ Pakistani/ Bangladeshi**	**Mixed ethnicity**	**All[2]**	p for ethnic differ-ence
Denominator (number of partnerships reported)[1]	*1037*	*404*	*245*	*443*	*189*	*329*	*2647*	
**Partnership type** at most recent sex with the partner								0.011
Married	3.4%	4.2%	8.6%	3.2%	12.7%	3.6%	4.6%	
Committed relationship but not married	39.4%	37.6%	42.4%	45.8%	41.3%	43.8%	41.2%	
Regular partners (but not in a committed relationship)	26.2%	25.2%	29.4%	32.3%	22.2%	29.2%	27.5%	
Had recently met	23.0%	24.3%	16.7%	15.6%	18.0%	17.6%	20.3%	
Had just met	8.0%	8.7%	2.9%	3.2%	5.8%	5.8%	6.4%	
**Partnership type** at most recent sex with the partner – 3 categories								0.009
Committed<br>(Married+committed but unmarried)	444	169	125	217	102	156	1213	
	42.8%	41.8%	51.0%	49.0%	54.0%	47.4%	45.8%	
Uncommitted but regular<br>(Regular partners but not in a committed relationship)	272	102	72	143	42	96	727	
	26.2%	25.2%	29.4%	32.3%	22.2%	29.2%	27.5%	
Casual<br>(Recently met+just met)	321	133	48	83	45	77	707	
	31.0%	32.9%	19.6%	18.7%	23.8%	23.4%	26.7%	
**Partnership duration (at most recent sex)[3]**								
< 4 weeks	24.1%	26.3%	20.8%	23.1%	21.7%	25.2%	23.9%	0.050
1-6 months	39.8%	40.4%	40.7%	29.1%	38.3%	36.6%	37.6%	
>6 months	36.2%	33.3%	38.4%	47.8%	40.0%	38.3%	38.5%	
By partnership type at most recent sex:								
Committed								
< 4 weeks	6.9%	5.9%	12.0%	10.2%	8.7%	10.3%	8.5%	0.123

1-6 months	27.5%	29.6%	36.1%	19.4%	31.5%	30.1%	27.9%	
>6 months	65.6%	64.5%	51.9%	70.4%	59.8%	59.6%	63.7%	
Uncommitted but regular								
< 4 weeks	11.4%	17.8%	13.1%	18.4%	10.5%	21.3%	15.0%	0.105
1-6 months	57.9%	61.6%	45.9%	40.8%	63.2%	46.3%	52.4%	
>6 months	30.7%	20.5%	41.0%	40.8%	26.3%	32.5%	32.6%	
Casual								
< 4 weeks	56.4%	56.1%	50.0%	64.5%	59.1%	57.5%	57.1%	0.403
1-6 months	41.3%	41.5%	47.7%	34.2%	31.8%	37.0%	39.8%	
>6 months	2.3%	2.4%	2.3%	1.3%	9.1%	5.5%	3.0%	
**Likelihood of sex again: Yes/probably[4]**	60.5%	66.1%	66.0%	69.1%	64.6%	64.4%	64.1%	0.093
By partnership type at most recent sex:								
Committed	84.0%	92.3%	81.3%	82.2%	86.9%	86.2%	85.1%	0.178
Uncommitted but regular	64.6%	65.6%	64.8%	65.4%	53.8%	55.1%	63.1%	0.467
Casual	23.9%	32.0%	27.7%	43.2%	21.4%	31.5%	28.7%	0.010
**Non-use of condom at most recent sex**	68.6%	62.2%	69.0%	67.5%	61.5%	67.2%	66.8%	0.275
By partnership type at most recent sex:								
Committed	76.9%	74.4%	74.0%	74.8%	66.7%	74.7%	74.7%	0.417
Uncommitted but regular	67.0%	58.0%	68.1%	64.8%	51.2%	64.9%	64.2%	0.277
Casual	58.7%	49.6%	57.4%	55.7%	60.5%	55.3%	56.3%	0.526
**How met partner[4]**								0.004
school/college/university/work	30.0%	23.7%	24.0%	23.5%	33.5%	24.8%	27.0%	
social venue	20.3%	28.3%	23.6%	24.4%	18.6%	20.2%	22.4%	
through friends/family	23.7%	19.8%	34.6%	30.0%	25.5%	32.2%	26.3%	
Online including internet dating	19.1%	21.0%	13.0%	12.2%	14.9%	14.7%	16.8%	
Other, inc. travelling, commercial sex	6.8%	7.3%	4.9%	9.9%	7.4%	8.0%	7.4%	

Notes for Supplementary Table 2B: [1]Denominator is partnerships reported by participants (SHC attendees) identifying as female, and who reported only male partners in the past year.

Aicken CRH, *et al. Sex Transm Infect* 2020; 96:283–292. doi: 10.1136/sextrans-2018-053739

[2]Excludes Other Asian/Chinese/Arab/Other.
[3]Estimated from questions about recency of first, and of most recent sex.
[4]Alternative responses included 'I don't know' as well as 'no' and 'probably not'.
[5]Categories in the table are based on a larger number of response options, as follows: school/college/university/work: 'At school', 'At college/university', 'At work (or through work)'; social venue/public place/neighbour: 'In a pub, bar, night club, disco, or dance', 'Through a sports club, faith group, or other organisation', 'Neighbour/lived locally/flat share', 'In a public place (e.g. park, café, shop, public transport)'; through friends/family: 'Introduced by friends or family', 'Had always known each other (e.g. as family friends)', 'Arranged marriage'; online, including internet dating: 'Internet dating website', 'Facebook', 'Twitter', 'Instagram', 'Pandora', 'Whatsapp', 'Other social media websites', 'Online but not through dating website or social media'; other: 'On holiday or while travelling', 'Other dating agency/personal ads', 'Partner was a sex worker', 'Partner was my client', 'Other'.

Aicken CRH, *et al. Sex Transm Infect* 2020; 96:283–292. doi: 10.1136/sextrans-2018-053739

Supplementary Table 3A: Age- and ethnic-mixing in men's opposite-sex partnerships in the past 3 months, by ethnic group

	**White British/ Irish**	**White other**	**Black African**	**Black Caribbean**	**Indian/ Pakistani/ Bangladeshi**	**Mixed ethnicity**	**All[2]**	p for ethnic differ-ence
Denominator (number of partnerships reported)[1]	*689*	*203*	*249*	*324*	*121*	*173*	*1759*	
Median age difference (IQR) (Man's age minus woman's age)[3]	1 (-1 to 4)	2 (-1 to 4)	2 (0 to 5)	2 (0 to 4)	2 (0 to 5)	2 (0 to 4)	2 (0 to 4)	
% of partnerships with age-mixing (≥5 yrs age difference)	29.0%	33.1%	36.3%	29.2%	33.9%	28.7%	30.8%	0.382
By partnership type at most recent sex:								
Committed	23.1%	24.0%	37.1%	35.9%	28.6%	17.9%	27.6%	0.285
Uncommitted but regular	30.3%	31.6%	31.0%	31.4%	19.0%	37.2%	31.0%	0.689
Casual	31.5%	36.1%	36.4%	21.8%	42.1%	28.9%	31.7%	0.198
% of partnerships with man ≥5yrs older than woman	21.7%	24.6%	27.8%	24.2%	25.7%	22.5%	23.7%	0.590
By partnership type at most recent sex:								
Committed	17.8%	18.0%	32.9%	29.5%	25.0%	15.4%	22.6%	0.264
Uncommitted but regular	22.7%	21.1%	23.9%	25.5%	14.3%	32.6%	24.1%	0.600
Casual	23.4%	26.5%	22.7%	18.2%	29.8%	21.1%	23.2%	0.655
% of partnerships with ethnic-mixing[4]	35.1%	50.5%	60.9%	67.3%	58.0%	86.2%	52.8%	<0.001
By partnership type at most recent sex:								
Committed	26.4%	41.5%	49.3%	57.7%	32.1%	82.1%	42.6%	<0.001
Uncommitted but regular	37.7%	48.9%	60.0%	64.2%	63.6%	78.3%	54.9%	0.001
Casual	38.9%	59.3%	73.3%	78.0%	67.8%	93.2%	58.3%	<0.001
Partner's ethnic group								<0.001
White British/Irish	64.9%	29.4%	20.2%	26.7%	17.9%	44.0%	42.4%	
White other	18.5%	49.5%	8.8%	5.7%	17.9%	18.2%	18.3%	
Black African	2.1%	2.1%	39.1%	8.3%	1.8%	2.5%	8.5%	
Black Caribbean	2.4%	1.5%	13.4%	32.7%	1.8%	7.5%	9.8%	

Aicken CRH, *et al. Sex Transm Infect* 2020; 96:283–292. doi: 10.1136/sextrans-2018-053739

Indian/Pakistani/Bangladeshi	2.0%	2.6%	2.5%	3.7%	42.0%	3.8%	5.3%	
Mixed	2.4%	3.6%	9.7%	15.7%	0.0%	13.8%	6.9%	
Other Asian/Chinese/Arab/other	7.8%	11.3%	6.3%	7.3%	18.8%	10.1%	8.9%	

Notes for Supplementary Table 3A: [1]Denominators: partnerships reported by participants (SHC attendees) identifying as male, and who reported only female partners in the past year.

[2]Excludes Other Asian/Chinese/Arab/Other.

[3]I.e. if a positive value, the man is older than the woman, and if a negative value, the woman is older than the man.

[4]Defined as partners of different ethnic groups, based on the broad ethnicity categories used in this paper, and including *partners* of Other Asian/Chinese/Arab/Other ethnic origin.

Aicken CRH, *et al. Sex Transm Infect* 2020; 96:283–292. doi: 10.1136/sextrans-2018-053739

Supplementary Table 3B: Age- and ethnic-mixing in women's opposite-sex partnerships in the past 3 months, by ethnic group

	**White British/ Irish**	**White other**	**Black African**	**Black Caribbean**	**Indian/ Pakistani/ Bangladeshi**	**Mixed ethnicity**	**All[2]**	p for ethnic differ-ence
Denominator (number of partnerships reported)[1]	*1041*	*411*	*250*	*449*	*190*	*331*	*2672*	
Median age difference (IQR) (Man's age minus woman's age)[3]	1 (0 to 4)	2 (0 to 5)	2 (0 to 5)	2 (0 to 5)	1 (0 to 4)	2 (0 to 4)	1 (0 to 4)	
% of partnerships with age-mixing (≥5 yrs age difference)	25.5%	34.1%	31.9%	31.6%	25.8%	28.4%	28.8%	0.064
By partnership type at most recent sex:								
Committed	23.8%	36.1%	31.1%	32.7%	25.5%	30.7%	28.8%	0.073
Uncommitted but regular	25.3%	31.6%	34.8%	26.7%	24.4%	28.6%	27.7%	0.448
Casual	27.8%	31.2%	31.0%	36.5%	28.6%	24.0%	29.2%	0.760
% of partnerships with man ≥5yrs older than woman	20.4%	26.4%	28.0%	25.7%	20.9%	24.6%	23.4%	0.143
By partnership type at most recent sex:								
Committed	20.3%	29.1%	28.7%	26.9%	20.4%	26.0%	24.3%	0.169
Uncommitted but regular	19.2%	26.5%	28.8%	22.2%	19.5%	27.5%	22.8%	0.495
Casual	21.9%	22.4%	26.2%	27.0%	23.8%	18.7%	22.6%	0.830
% of partnerships with ethnic-mixing[4]	32.1%	59.9%	28.9%	39.6%	49.4%	79.3%	44.1%	<0.001
By partnership type at most recent sex:								
Committed	26.5%	54.7%	26.6%	33.5%	38.4%	76.9%	38.8%	<0.001
Uncommitted but regular	35.5%	70.0%	29.2%	36.8%	53.7%	85.4%	47.4%	<0.001
Casual	37.5%	60.6%	33.3%	58.5%	74.4%	77.6%	50.3%	0.001
Partner's ethnic group								<0.001
White British/Irish	67.9%	32.5%	4.5%	7.4%	12.8%	22.0%	36.9%	
White other	10.4%	40.1%	1.2%	1.6%	5.0%	6.7%	11.8%	
Black African	4.6%	5.8%	71.1%	18.5%	3.9%	10.3%	14.2%	
Black Caribbean	7.0%	6.1%	16.7%	60.4%	12.8%	32.3%	20.2%	
Indian/Pakistani/Bangladeshi	2.2%	3.3%	0.4%	0.7%	50.6%	2.3%	5.4%	

Mixed	5.0%	4.6%	6.1%	7.2%	7.8%	20.7%	7.4%	
Other Asian/Chinese/Arab/other	3.0%	7.6%	0.0%	4.2%	7.2%	5.7%	4.2%	

Notes for Supplementary Table 3B: [1]Denominators reported by participants (SHC attendees) identifying as female, and who reported only male partners in the past year.

[2]Excludes Other Asian/Chinese/Arab/Other.

[3]I.e. if a positive value, the man is older than the woman, and if a negative value, the woman is older than the man.

[4]Defined as partners of different ethnic groups, based on the broad ethnicity categories used in this paper.

Aicken CRH, *et al. Sex Transm Infect* 2020; 96:283–292. doi: 10.1136/sextrans-2018-053739

Supplementary Table 4A: The role of sexual mixing and partnership type in explaining inter-ethnic differences in acute STI diagnosis: men

	**White British/ Irish**	**White other**	**Black African**	**Black Caribbean**	**Indian/ Pakistani/ Bangladeshi**	**Mixed ethnicity**	**All[2]**	p for ethnic difference
Denominator (participants)[1]	*364*	*108*	*125*	*149*	*71*	*83*	*900*	
**'Acute STI':** Bacterial STI or TV diagnosis/es within +/- 6 weeks of clinic attendance (95% CI)	20.1% (15.9-25.0%)	21.3% (14.9-29.5%)	20.8% (15.5-27.2%)	26.8% (15.2-42.9%)	9.9% (3.1-27.2%)	33.7% (24.8-44.0%)	21.9% (18.4-25.9%)	0.094
Unadjusted OR (95% CI)	1 -	1.08 (0.72-1.61)	1.05 (0.71-1.54)	1.46 (0.69-3.10)	0.44 (0.13-1.50)	2.03 (1.23-3.35)	- -	0.094
aOR1: Adjusted for age and number of recent partners[3] (95% CI)	1 -	1.11 (0.75-1.66)	1.02 (0.70-1.47)	1.39 (0.66-2.91)	0.48 (0.14-1.65)	2.00 (1.25-3.20)	- -	0.069
aOR2: Adjusted for the above variables, and sexual mixing and partnership type[4] (95% CI)	1 -	1.09 (0.82-1.47)	1.06 (0.72-1.57)	1.43 (0.56-3.68)	0.49 (0.13-1.85)	2.13 (1.40-3.25)	- -	0.025

Notes for Supplementary Table 4A: [1]Denominators: participants (SHC attendees) identifying as male, and who reported only female partners in the past year.

[2]Excludes Other Asian/Chinese/Arab/Other.

[3]Adjusted for: age as a continuous variable, and number of opposite-sex partners in the past 3 months (0, 1, 2+).

[4]Adjusted for: age as a continuous variable, number of opposite-sex partners in the past 3 months (0, 1, 2+), and the following (all derived from questions about the (up to) 3 most recent partners within the past 3 months): any committed partners within the past 3 months, any uncommitted regular partners within the past 3 months, any casual partners within the past 3 months; and the following sexual mixing variables: any age-mixing* among partners within the past 3 months, any ethnic-mixing among partners within the past 3 months. (*We also repeated this using the variable for age-mixing with man older. Results were very similar.)

Aicken CRH, *et al. Sex Transm Infect* 2020; 96:283–292. doi: 10.1136/sextrans-2018-053739

Supplementary Table 4B: The role of sexual mixing and partnership type in explaining inter-ethnic differences in acute STI diagnosis: women

	**White British/Irish**	**White other**	**Black African**	**Black Caribbean**	**Indian/ Pakistani/ Bangladeshi**	**Mixed ethnicity**	**All[2]**	p for ethnic difference
Denominator (participants)[1]	*664*	*261*	*184*	*319*	*129*	*225*	*1782*	
**'Acute STI':** Bacterial STI or TV diagnosis/es within +/- 6 weeks of clinic attendance (95% CI)	7.7% (5.3-10.9%)	11.9% (9.1-15.4%)	13.6% (9.5-19.0%)	16.0% (9.9-24.8%)	7.0% (4.6-10.4%)	15.6% (11.4-20.8%)	11.3% (8.7-14.7%)	0.003
Unadjusted OR (95% CI)	1 -	1.62 (1.23-2.14)	1.89 (1.12-3.20)	2.29 (1.24-4.24)	0.90 (0.58-1.40)	2.21 (1.48-3.31)	- -	<0.001
aOR1: Adjusted for age and number of recent partners[3] (95% CI)	1 -	1.82 (1.46-2.25)	1.75 (1.10-2.77)	2.36 (1.33-4.19)	0.92 (0.59-1.45)	2.12 (1.50-3.00)	- -	<0.001
aOR2: Adjusted for the above variables, and sexual mixing and partnership type[4] (95% CI)	1 -	1.83 (1.33-2.53)	1.63 (0.90-2.94)	2.23 (1.12-4.43)	1.00 (0.61-1.66)	2.05 (1.42-2.97)	- -	<0.001

Notes for Supplementary Table 4B: [1]Denominator is participants (SHC attendees) identifying as female, and who reported only male partners in the past year.

[2]Excludes Other Asian/Chinese/Arab/Other.

[3]Adjusted for: age as a continuous variable, and number of opposite-sex partners in the past 3 months (0, 1, 2+).

[4]Adjusted for: age as a continuous variable, number of opposite-sex partners in the past 3 months (0, 1, 2+), and the following (all derived from questions about the (up to) 3 most recent partners within the past 3 months): any committed partners within the past 3 months, any uncommitted regular partners within the past 3 months, any casual partners within the past 3 months; and the following sexual mixing variables: any age-mixing* among partners within the past 3 months, any ethnic-mixing among partners within the past 3 months. (*We repeated this analysis replacing 'any age-mixing' with a variable for any age-mixing in which the man was ≥5 years older. Results were very similar.)

Aicken CRH, *et al. Sex Transm Infect* 2020; 96:283–292. doi: 10.1136/sextrans-2018-053739

Supplementary Table 5: Illustrating how adjusted odds ratios for acute STI diagnosis (comparing ethnic groups and gender) differ very little depending which age-mixing variable is used, in Table 4's multivariable analysis

		Men		Women		Comparisons by gender			
						Among BC attendees		Among WBI attendees	
	Age mixing variable	BC compared to WBI	p	BC compared to WBI	p	men compared to women	p	men compared to women	p
aOR2, as per Table 4 (95% CI)	*Any age-mixing reported within past 3 months, with age difference between partners ≥5 years*	1.53 (0.57-4.06)	0.367	2.15 (1.06-4.38)	0.037	2.01 (1.21-3.33)	0.011	2.70 (1.61-4.51)	<0.001
aOR2, substituting 'age-mixing with man older' for 'any age mixing' (95%CI)	*Any age-mixing reported within past 3 months, with man ≥5 years older than woman*	1.53 (0.58-4.04)	0.364	2.14 (1.05-4.37)	0.038	1.99 (1.22-3.24)	0.009	2.70 (1.61-4.53)	0.001

Aicken CRH, *et al. Sex Transm Infect* 2020; 96:283–292. doi: 10.1136/sextrans-2018-053739
