# Supplementary material for: Ethnic variations in sexual partnerships and mixing, and their association with STI diagnosis: findings from a cross-sectional biobehavioural survey of attendees of sexual health clinics across England
Source: Sex Transm Infect. 2019 Aug 17;96(4):283–92. doi: 10.1136/sextrans-2018-053739 (PMC7279208; doi:10.1136/sextrans-2018-053739)
Supplement: Supplementary data [file sextrans-2018-053739supp002.pdf]

Supplementary Figure 1: Flowchart of bio-behavioural survey participation, and inclusion/exclusion

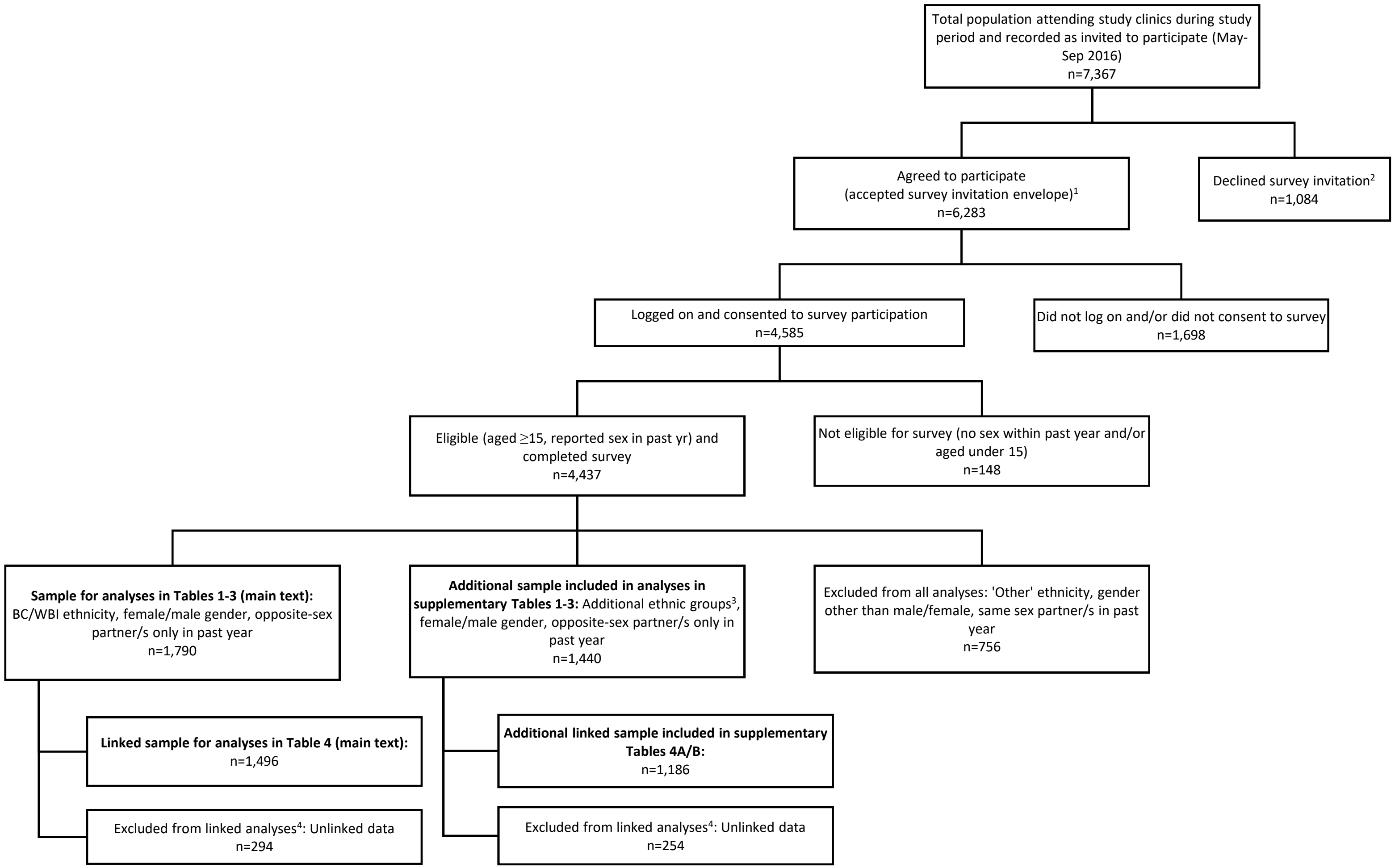

[1]Our survey, the Bio-Behavioural Enhanced Surveillance Tool (BBEST) had two versions, reflecting the HPRU's two populations of interest: Black Caribbean (BC) SHC attendees, and men who have sex with men (MSM) SHC attendees. Our analysis concerns the version of BBEST which targeted BC attendees (although it was open to other ethnicities and sexual orientations), through 16 purposively-selected clinics, as described in Methods. Because of how the survey was implemented, figures for both versions of the survey are included in this flowchart. (In three of the 16 clinics, MSM, of all ethnicities, were routed to the MSM version of the survey, and an additional 3 clinics recruited only to the MSM version of the survey.)
Of the 7367 attendees invited, 85.28% (6283) agreed to participate. Of these, 72.97% (4585/6283) logged in and consented to survey participation.(Wayal et al., 2018)
[2]Recruitment differed between clinics, related to staff's commitment to and enthusiasm for the research, and the availability of resources to support research; in some clinics the survey invitation was not consistently offered to all participants.(Wayal et al., 2018)
[3]Additional ethnic groups included in supplementary table analyses are: Black African, White Other, Indian/Pakistani/Bangladeshi, Mixed ethnicity. Supplementary tables also include participants of Black Caribbean and White British/Irish ethnicities.
[4]Survey data from some participants were not linked to clinical data obtained from GUMCAD. A breakdown of this process is as follows: among the n=4437 participants who were eligible for and completed the survey, 91.2% provided additional consent to linkage of their survey data with an extract of their clinical data (4046/4437). Of these, data linkage was achieved for 88.9% (3596/4046), but not for 11.1% (450/4046). Among the latter group, who consented to data linkage but for whom linkage was not achieved, 34.7% (156/450) could not be linked because clinic staff did not record either the participant's clinic number or the unique study passcode.
